# Functions of lncRNA HOTAIR in lung cancer

**DOI:** 10.1186/s13045-014-0090-4

**Published:** 2014-12-10

**Authors:** Gregory Loewen, Janarthanan Jayawickramarajah, Ying Zhuo, Bin Shan

**Affiliations:** Providence Regional Cancer Center, 105 W. 8th Avenue, Spokane, 99204 WA USA; Department of Chemistry, Tulane University, 2015 Percival Stern Hall, New Orleans, 70118 LA USA; Kadlec Regional Medical Center, 888 Swift Boulevard, Richland, 99352 WA USA; College of Medical Sciences, Washington State University Spokane, 412 E. Spokane Falls Boulevard, Spokane, 99202 WA USA

**Keywords:** lncRNA, HOTAIR, Lung cancer, PRC2, Metastasis

## Abstract

Long non-coding RNAs (lncRNAs) govern fundamental biochemical and cellular processes. lncRNA HOX transcript antisense RNA (HOTAIR) represses gene expression through recruitment of chromatin modifiers. The expression of HOTAIR is elevated in lung cancer and correlates with metastasis and poor prognosis. Moreover, HOTAIR promotes proliferation, survival, invasion, metastasis, and drug resistance in lung cancer cells. Here we review the molecular mechanisms underlying HOTAIR-mediated aggressive phenotypes of lung cancer. We also discuss HOTAIR’s potential in diagnosis and treatment of lung cancer, as well as the challenges of exploiting HOTAIR for intervention of lung cancer.

**Electronic supplementary material:**

The online version of this article (doi:10.1186/s13045-014-0090-4) contains supplementary material, which is available to authorized users.

## lncRNAs as novel master regulators of lung cancer

A surprising discovery of the ENCODE project is that 87.3% of the human genome is actively transcribed although only < 3% of the human genome encodes proteins [1]. One family of the non protein-coding RNAs is operationally defined as long non-coding RNAs (lncRNAs) based on their length > 200 nucleotides [2]. As published in GENCODE v7 (2012), the lncRNA catalogue comprises 9277 manually annotated lncRNA genes that produce 14,880 transcripts [3]. lncRNAs regulate fundamental biochemical and cellular processes, such as gene expression, RNA splicing, and ligand-receptor engagement, which mediates pathogenesis of benign and malignant respiratory disorders [4],[5].

lncRNAs have emerged as novel master regulators of initiation, progression, and response to therapy in a wide variety of solid tumors and hematological malignancies [6],[7]. Hundreds of IncRNAs have been associated with lung cancer through gene expression microarrays and massively parallel RNA sequencing of tumor tissues and paired adjacent non-tumor tissues in the lung [8]-[11]. As of September 2014, a PubMed search using lncRNA and lung cancer as key words yielded more than a dozen of lncRNAs that have been individually investigated in lung cancer (Table 1) [8],[12]-[38]. Despite their largely descriptive and correlative nature, these reports highlight a critical role of lncRNAs in lung cancer. The investigated lncRNAs regulate critical cellular processes in lung cancer, such as proliferation, invasion, and survival (Table 1). Moreover, dysregulated expression of these lncRNAs is correlated with metastasis, advanced pathological stages, and poor prognosis in patients with lung cancer (Table 1).

**Table 1:** **Lung cancer-associated lncRNAs**

lncRNA	Intersecting molecules and pathways	Cell processes	Associated clinical features
AK126698	Reduces NKD2, activates β-catenin [12]	Anti-apoptosis, resistance to cisplatin [12]	Unknown
CARLo-5	Unknown	Cell cycle, proliferation, invasion, EMT [13]	↑ in NSCLC, lymph node metastasis, poor survival [13]
CCAT2	Unknown	Proliferation, migration, invasion [14]	↑ in LAC, lymph node metastasis [14]
H19	Induced by cigarette smoke [15],[16]	Unknown	↑ in NSCLC [17], poor survival [18]
HOTAIR	Induced by Col-1 [19]. Affects expression of gelatinases [20]. Represses cell-adhesion genes [21], p21^waf1^[22], and HOXA5 [23]	Proliferation, migration invasion [20],[21],[23]; resistance to cisplatin in vitro & in vivo [22]	↑ in NSCLC, lymph node and brain metastasis, poor survival [19],[20],[23],[24]. ↑ in cisplatin-refractory LAC [22]. ↑ in SCLC, lymphatic invasion, relapse [21]
LCAL1	Unknown	Proliferation [8]	↑ in NSCLC [8]
MALAT1	Affects expression of Bcl-2 [25] and metastasis related genes [26]	EMT [27], tumor growth in vivo [26], survival [25]	↑ in NSCLC, brain metastasis, poor survival [25],[27]. ↑in periphery blood of NSCLC [28]
MVIH	Affects expression of MMP-2/-9 [29]	Proliferation & invasion [29]	↑ in LAC and LSCC, advanced TNM stage, lymph node metastasis, poor prognosis [29]
SCAL1	Induced by cigarette smoke and NRF2 [30]	Protection against oxidative stress [30]	↑ NSCLC [8],[30]
SOX2ot	Affects expression of EZH2 [31]	Cell cycle, proliferation [31]	↑ in LSCC, poor survival [31]
ZXF1	Antisense to ACTA2 [32]	Migration & invasion [32]	↑ in LAC, lymph node metastasis, advanced TNM stage, poor survival [32]
BANCR	Inhibits the expression of EMT markers [33]	Induces apoptosis, inhibits EMT, migration, invasion, metastasis in vivo [33]	↓ in LAC and LSCC, lymph node metastasis, advanced TNM stage, poor survival [33]
GAS6-AS1	Antisense to and represses expression of GAS6 [34]	Unknown	↓ in NSCLC, advanced TNM stage, poor survival [34]
MEG3	Induces p53 [35]	Inhibits proliferation & growth in vivo, pro-apoptosis [35]	↓ in NSCLC, advanced TNM stage, poor survival [35]
SPRY4-IT1	Intronic to SPRY4, silenced by EZH2 [36]	Inhibits invasion, growth & metastasis in vivo, induces apoptosis [36]	↓ in NSCLC, pathological stage, lymph node metastasis [36]
TARID	Activates TCF21 via GADD45A [37]	Unknown	↓ in LAC and LSCC [37]
TUG1	Induced by p53, represses HOXB7 via PRC2 [38]	Inhibits proliferation & growth in vivo [38]	↓ in NSCLC, advanced TNM stage, poor survival [38]

A summary of the lung cancer-associated lncRNAs and the molecular pathways, cell processes, and clinical features that are linked to these lncRNAs. See text for details. CARLo-5: Cancer-associated region long non-coding RNA; CCAT2: colon cancer-associated transcript 2; HOTAIR: HOX transcript antisense RNA; LCAL1: lung cancer associated lncRNA 1; MALAT1: Metastasis associated in lung adenocarcinoma transcript 1; MVIH: microvascular invasion in hepatocellular carcinoma; SCAL1: smoke and cancer-associated lncRNA-1; SOX2ot: Sox2 overlapping transcript; BANCR: BRAF activated non-coding RNA; GAS6-AS1: GAS6 antisense RNA 1; MEG3: Maternally expressed gene 3; SPRY4-IT1: SPRY4 intronic transcript 1; TARID: TCF21 antisense RNA inducing demethylation; TUG1: taurine-upregulated gene 1; NSCLC: non-small cell lung cancer; SCLC: small cell lung cancer; LAC: lung adenocarcinoma; LSCC: lung squamous cell carcinoma.

↑ and ↓ indicate increase and decrease, respectively.

The lncRNA HOX Transcript Antisense RNA (HOTAIR) has attracted intense investigation in lung cancer (Table 1) [19]-[24],[39]. Herein we review the literature of HOTAIR in lung cancer with an emphasis on the molecular mechanisms underlying its regulation of lung cancer. To obtain comprehensive insight of HOTAIR in lung cancer, we integrate mechanistic studies of HOTAIR in other types of cancer in our review.

### Discovery of the HOTAIR gene

**Figure 1:**
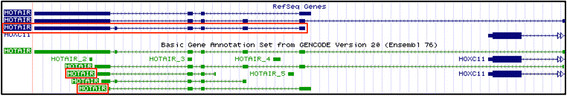
**Isoforms of human HOTAIR transcripts.** The USCS Genome Browser track of the human HOTAIR gene is used to illustrate isoforms of the human HOTAIR transcript [47]. The principal transcript RefSeq NR_003716 and two multi-exon HOTAIR variants in the GENCODE catalogue that lack the PRC2-interacting domain and the LSD1-interacting domain are marked by red open rectangles. See text for details.

**Figure 2:**
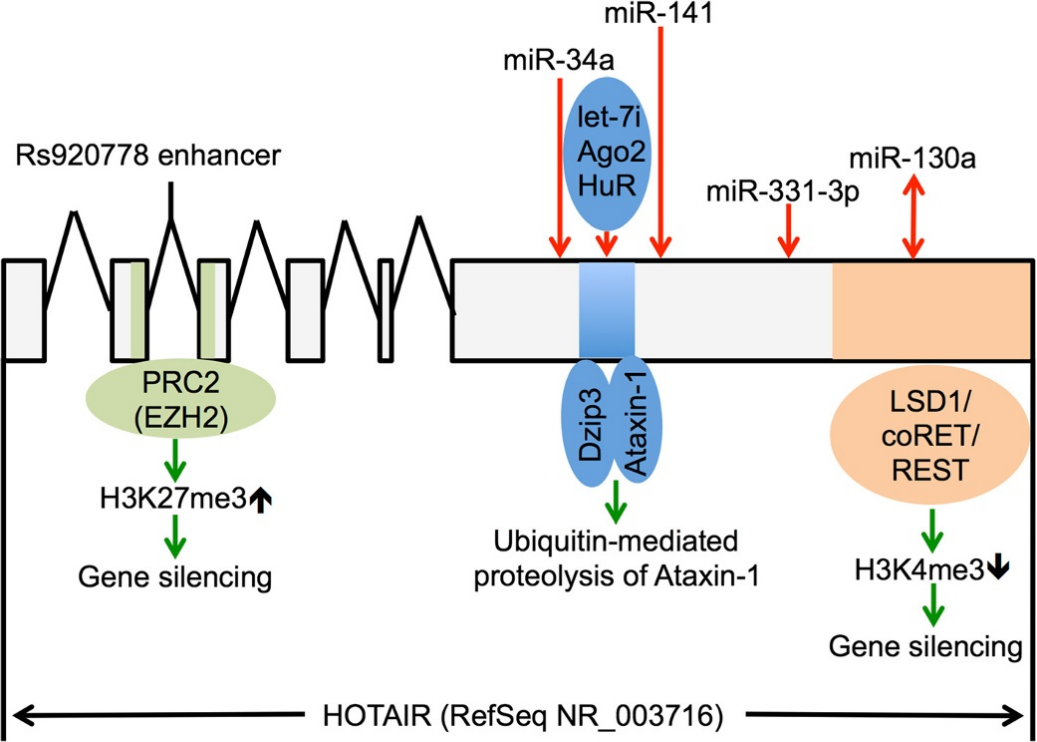
**Molecular mechanisms of the tumor-promoting actions of HOTAIR.** The interactions between HOTAIR and its partners are summarized. The length of each exon and positions of the interacting region for each partner are proportional to their length and positions in the principal transcript RefSeq NR_003716 of the human HOTAIR gene. The introns are not drawn proportionally to their length. A green arrow indicates positive regulation of the processes or substrates targeted by the arrow. A red arrow indicates negative regulation of the targeted processes or substrates by the arrow. A red bi-directional arrow is used to illustrate the reciprocal negative regulation between HOTAIR and miR-130a. HOTAIR’s interaction with E3 ubiquitin ligase Mex3b and its substrate Snurportin-1 is not included in the figure because the interaction is mediated through the region that overlaps with the Dzip3-Ataxin-1 interacting domain in HOTAIR. See text for details. PRC2: Polycomb Repressive Complex 2; EZH2: enhancer of zeste homolog 2; HuR: Human antigen R; LSD1: Lysine-Specific Demethylase 1.

HOTAIR was discovered by Howard Chang’s group as a lncRNA that recruits Polycomb Repressive Complex 2 (PRC2), a transcriptional co-repressor, to repress the expression of the homeobox gene D cluster (HOXD) [39]. The human HOTAIR gene resides within the intergenic region between HOXC11 and HOXC12 in the HOXC cluster on chromosome 12. The HOTAIR gene is transcribed in an antisense direction relative to its flanking HOXC11 and HOXC12 genes. Its principal transcript (RefSeq NR_003716) is a 2364 bp RNA transcribed from a 6449 bp gene locus and composed of 6 exons (Figure 1, marked by an red open rectangle). An 89 bp fragment in the 5′ end of HOTAIR (221–300 bp in RefSeq NR_003716) binds to PRC2, and a 646 bp fragment in its 3′ end binds to the LSD1/CoREST/REST complex (Figure 2) [40],[41]. PRC2 contains Enhancer of Zeste Homolog 2 (EZH2), a histone methyltransferase that marks a gene for transcriptional repression via tri-methylation of histone H3 Lys27 (H3K27me3) [42]. HOTAIR appears to bind to GA-rich motifs in the genome to nucleate broad domains of PRC2 occupancy and consequent H3K27me3 [43]. The LSD1/CoREST/REST complex contains Lysine-Specific Demethylase 1 (LSD1), a histone demethylase that inactivates gene expression via demethylation of the di-methylated histone H3 Lys4 (H3K4me2), a histone modification that is critical for transcriptional activation [44]. Methylation of C1683 in HOTAIR’s principal transcript (RefSeq NR_003716) at the boundary of the LSD1-binding motif is thought to be critical for the HOTAIR-LSD1 physical interaction [45]. Acting as a bridging scaffold for PRC2 and LSD1/CoREST/REST, HOTAIR represses gene expression by coupling an increase of the repression code H3K27me3 with a decrease of the activation code tri-methylation of histone H3 lysine 4 (H3K4me3) on its target promoters [41]. In accordance, deletion of the mouse Hotair gene results in de-repression of the HOXD cluster that is coupled with decreased occupancy of H3K27me3 and increased occupancy of H3K4me3 on the HOXD gene promoters due to a loss of HOTAIR-mediated recruitment of PRC2 and LSD1 [46]. Consequently, HOTAIR null mice exhibit homeotic transformation of the spine and malformation of metacarpal-carpal bones [46].

The human HOTAIR gene can be transcribed into several variants via alternative splicing as illustrated in the GRCh38/hg38 Assembly on UCSC Genome Browser (Figure 1). The RefSeq catalogue includes three HOTAIR variants (Figure 1). The GENCODE v20 catalogue includes nine HOTAIR variants and four of them are single exon transcripts (Figure 1). A recent study using a targeted RNA capture and sequencing strategy identified six major HOTAIR splicing variants and proposed one alternative splice site, when active, can eliminate the PRC2 binding domain [48]. Consistently, two multi-exon HOTAIR variants in the GENCODE catalogue lack the PRC2-interacting domain and the LSD1-interacting domain (Figure 1, marked by red open rectangles). It is a worthy cause to determine whether alternative splicing of HOTAIR is regulated in any physiological or pathological context and whether the splicing variants exert different functions due to their different structures.

Since its first link to metastasis in breast cancer, elevated expression of HOTAIR has been reported in at least 16 types of malignancies [19],[22]-[24],[49]-[82]. Dysregulated expression of HOTAIR has not yet been reported in hematological malignancies, although the protein-coding HOX genes play a critical role in those disorders [83].

### Expression of HOTAIR in lung cancer

HOTAIR exhibits significantly higher expression in the tumor tissue than the adjacent non-tumor tissue in patients with small cell lung cancer (SCLC) and non-small cell lung cancer (NSCLC) (Table 1) [19]-[24]. In SCLC, elevated expression of HOTAIR is linked to lymphatic invasion and relapse (Table 1) [21]. In NSCLC, elevated expression of HOTAIR is linked to lymph node metastasis and poor survival in patients with lung adenocarcinoma (LAC) and squamous cell carcinoma (LSCC) (Table 1) [19],[20],[22]-[24]. Moreover, elevated expression of HOTAIR is correlated with brain metastasis in NSCLC [24].

It remains unknown whether elevated expression of HOTAIR in lung cancer is caused by genetic alterations, such as amplification, deletion, or point mutations. One recent study reported that the human HOTAIR gene harbors an enhancer-like region between +1719 bp and +2353 bp downstream of its transcription start site in intron 2 [84]. The enhancer contains a risk SNP rs920778 for esophageal squamous cell carcinoma, and the rs920778T allele containing the enhancer drives higher expression of a reporter gene than the rs920778C allele (Figure 2) [84]. More importantly, the rs920778TT allele is correlated with higher expression of HOTAIR in the esophageal tissue than the rs920778CC allele, and the HOTAIR rs920778TT carriers are at a higher risk of esophageal squamous cell carcinoma than the HOTAIR rs920778CC carriers [84].

One emerging mechanism underlying up-regulation of HOTAIR in cancer cells is direct transcriptional activation of HOTAIR by classical oncogenes. For instance, HOTAIR is transcriptionally activated by the oncogene Myc through an E-box located at 1053 bp upstream of the transcription start site of the human HOTAIR gene in gallbladder cancer cells [85]. Because Myc is also a well-documented oncogene in lung cancer, this mechanism needs to be explored in lung cancer [86].

Transcriptional up-regulation of the human HOTAIR gene in cancer involves epigenetic mechanisms. An intriguing observation in breast cancer tissues is that increased DNA methylation in an intergenic CpG island located between HOXC12 and HOTAIR is positively correlated with HOTAIR expression in breast cancer [69]. It is proposed by the authors that the methylated intergenic CpG island acts as a barrier to prevent repressive heterochromatin from spreading from the HOXC12 gene into the neighboring HOTAIR gene [69]. On the other hand no CpG insland is predicted in the human HOTAIR promoter (2 kb upstream of HOTAIR’s transcription start site) using MethPrimer [87]. Besides DNA methylation histone modifications regulate the expression of HOTAIR. In breast cancer cells, estradiol activates the expression of HOTAIR via recruitment of histone methyltransferases mixed lineage leukemia proteins (MLL) to the HOTAIR promoter [50]. Consequently, MLL poises the HOTAIR promoter for transcription via H3K4me3.

Similar to protein-coding genes, lncRNAs have emerged as targets of microRNAs in a base-pairing fashion [88]. In exon 6, the HOTAIR transcript harbors a target site for miR-34a (902–923 bp in RefSeq NR_003716) (Figure 2) [54]. miR-34a reduces the expression of HOTAIR and a reporter gene that is controlled by the miR-34a target site from HOTAIR in prostate cancer cells [54]. A target site for miR-141 is identified in exon 6 of the HOTAIR transcript (1287–1308 bp in RefSeq NR_003716) (Figure 2) [53]. miR-141 reduces the expression of HOTAIR and a reporter gene that is controlled by the miR-141 target site from HOTAIR in renal carcinoma cells [53]. It is noteworthy that miR-141 is a member of the miR-200 family, one of the most potent miRNA inhibitors of epithelial-mesenchymal transition (EMT), a pathological process that is promoted by HOTAIR in cancer [49],[89]. HOTAIR is also predicted to harbor a let-7i target site in its exon 6 (2120–2141 bp in RefSeq NR_003716) although its binding to let-7i has not been experimental validated (Figure 2) [90]. Nevertheless the RNA levels of HOTAIR can be reduced by overexpression of let-7i and increased by introduction of a let-7i-specific antagomir [90]. let-7i-mediated decay of HOTAIR appears to rely on formation of a hetero-tetramer that consists of HOTAIR, let-7i, Ago2, and a RNA binding protein human antigen R (HuR). The HuR binding domain in HOTAIR is mapped to exon 6 (~1,028–1,272 bp in RefSeq NR_003716). Although it remains unclear how let-7i, Ago2, and HuR coordinate decay of HOTAIR, HuR’s binding to HOTAIR appears to recruit the let-7i/Ago2 complex to HOTAIR for decay (Figure 2) [90]. In summary, the tumor suppressive miRNA-mediated decay of HOTAIR, although established in other cancer types, warrants further investigation in lung cancer because let-7, miR-34, and miR-141 act as critical tumor suppressors in lung cancer [91]-[94].

An intriguing phenomenon observed in the seminal study of HOTAIR in breast cancer is that established breast cancer cell lines exhibit a much lower expression of HOTAIR than breast cancer tissues [60]. This apparent discrepancy might be attributed to activation of HOTAIR expression by several metastasis-promoting signals that are aberrantly enriched in the tumor microenvironment but absent in routine cell culture. For instance, transforming growth factor-β1 (TGF-β1) activates the expression of HOTAIR in breast and colon cancer cells, and such an induction is required for acquisition of EMT and cancer stem cell phenotypes [49],[95]. Prolonged exposure of human breast cancer MCF-7 cells to tumor necrosis factor-α (TNF-α) induces the expression of HOTAIR and EMT [96]-[98]. Moreover, type 1 collagen transcriptionally up-regulates the expression of HOTAIR in lung adenocarcinoma cells [19]. Interestingly, all three stimuli are potent inducers of EMT in lung cancer cells and can up-regulate expression of several tumor-promoting miRNAs, such as miR-21 and the miR-17 ~ 92 cluster [95],[96],[99]-[102].

### Functions of HOTAIR in lung cancer

Elevated expression of HOTAIR is correlated with invasion, metastasis, and poor survival in patients with lung cancer (Table 1) [19]-[24]. In lung cancer cells HOTAIR regulates genes and signaling pathways that are pivotal to differentiation, proliferation, and invasion. Among the HOTAIR-regulated genes in lung cancer cells, HOXA5 is of particular interest because of its established roles in lung development and tumorigenesis [23]. HOXA5 is essential to morphogenesis of the embryonic respiratory tract and postnatal lung development [103]. Interestingly, HOXA5 is also down-regulated by another HOX cluster derived non-coding RNA, miR-196a, whose expression is inversely correlated with HOXA5 in lung cancer [104]. It is plausible that HOTAIR and miR-196a act in concert to repress the expression of HOXA5 and thereby promote dedifferentiation of lung epithelial cells during lung tumorigenesis. Another HOTAIR-repressed gene is p21^WAF1/CIP1^, a mediator of p53-induced growth arrest and apoptosis in response to DNA damage [22]. HOTAIR promotes proliferation, survival, and resistance to cisplatin through repression of p21^WAF1/CIP1^ in lung adenocarcinoma cells [22]. Thus HOTAIR can promote dedifferentiation and proliferation in lung cancer.

In addition to proliferative phenotype, HOTAIR mediates invasive phenotype of lung cancer cells through its promotion of EMT. EMT is defined as a series of events through which epithelial cells lose many of their epithelial characteristics and acquire property that is typical of mesenchymal cells, which leads to invasiveness and stemness of cancer cells [105]. During EMT, HOTAIR represses the expression of cell adhesion-related genes that are characteristic of epithelial cells in SCLC cells [21]. HOTAIR also mediates EMT via repression of EMT inhibitors. For instance, HOTAIR represses the expression of Wnt inhibitory factor 1 (WIF-1), an inhibitor of the Wnt/β-catenin pathway that mediates EMT in esophageal cancer cells [58]. In addition HOTAIR represses the expression of phosphatase and tensin homolog (PTEN), an inhibitor of EMT, in laryngeal squamous cell carcinoma cells [65]. Besides repression of EMT inhibitors, HOTAIR also mediates the expression of EMT effectors. For example, HOTAIR is required for the expression of matrix metalloproteinases that break down the extracellular matrix to pave the path for invasion in lung cancer cells [20],[59],[76],[79],[106]. Taken together, HOTAIR is induced by EMT stimuli, and such an induction in turn promotes the gene expression program that results in EMT.

The prevailing mechanism of HOTAIR-mediated regulation of cancer is that elevated expression of HOTAIR shifts PRC2-mediated gene repression from tumorigenic genes to tumor-suppressive genes [60],[61],[63],[64]. This mode of action is supported by studies on HOTAIR’s partners in lung cancer. The components of PRC2 are overexpressed in lung cancer and exert tumorigenic effects in lung cancer. EZH2 is overexpressed in SCLC and represses the expression of cell adhesion-related genes, which resembles the effects of overexpression of HOTAIR in SCLC cells [21],[107]. Another PRC2 component, SUZ12, promotes proliferation and metastasis of NSCLC cells via repression of E2F1, ROCK1, and ROBO1 [108]. Besides PRC2, HOTAIR may promote lung cancer through LSD1. LSD1 mediates proliferation and EMT in lung cancer cells, and its overexpression is associated with shorter overall survival of patients with SCLC and NSCLC [109],[110].

HOTAIR can potentially regulate lung cancer through physical interactions with E3 ubiquitin ligases and their corresponding substrates. For instance, E3 ubiquitin ligase Dzip3 and its substrate Ataxin-1 bind tandem to a ~250 nucleotide region in exon 6 (~1,028–1,272 bp in RefSeq NR_003716) through their respective RNA binding domains [90]. On the other hand, E3 ubiquitin ligase Mex3b and its substrate Snurportin-1 bind to HOTAIR in two far apart regions at ~125–250 bp and ~1,142–1,272 bp (RefSeq NR_003716), respectively [90]. Thus HOTAIR serves as an assembly scaffold that facilitates the interactions of the bound E3 ubiquitin ligases and their corresponding substrates, which leads to proteolysis of Ataxin-1 and Snurportin-1 [90]. Intriguingly, Ataxin-1, Snurportin-1, and HuR appear to compete for the same region in HOTAIR (~1,028–1,272 bp in RefSeq NR_003716) that mediates decay of HOTAIR upon HuR binding (Figure 2) [90]. It is plausible that HuR-mediated decay of HOTAIR and HOTAIR-mediated ubiquitination of Ataxin-1 and Snurportin-1 are mutually exclusive because of their competition for the same region in HOTAIR. The intertwining of HOTAIR decay and proteolysis may play a role in cell senescence. Induction of HOTAIR in senescent cells prevents premature senescence via interaction with Dzip3 and Mex3b and the consequent rapid proteolysis of Ataxin-1 and Snurportin-1 [90]. HOTAIR-mediated regulation of senescence is potentially important in lung cancer because evasion of senescence is proposed as a critical step in lung tumorigenesis [111]. Moreover, HOTAIR-mediated ubiquitination and degradation of Ataxin-1 is of particular interest to lung cancer because Ataxin-1 is essential to lung alveolization [112]. Thus HOTAIR may promote dedifferentiation of lung epithelial cells through two distinct mechanisms, i.e., transcriptional repression of HOXA5 and ubiquitin-mediated proteolysis of Ataxin-1 [23],[90].

An emerging theme in the non-coding RNA world is the crosstalk between miRNAs and lncRNAs [88]. As discussed above, the expression of HOTAIR is regulated by several tumor suppressive miRNAs, such as miR-34a and miR-141 in cancer cells (Figure 2) [53],[54]. On the other hand, HOTAIR antagonizes several tumor suppressive miRNAs. In gastric cancer cells, HOTAIR acts as a competitive endogenous RNA (ceRNA) to trap miR-331-3p through a complementary target site (1451–1471 bp in RefSeq NR_003716) and thereby increases the expression of the miR-331-3p-targeted oncogene HER2 (Figure 2) [68]. In gall bladder cancer, HOTAIR’s oncogenic activity requires its binding to and neutralization of miR-130a (1805–1826 bp in RefSeq NR_003716) (Figure 2) [85]. Reciprocally, miR-130a represses the expression of HOTAIR in a target site-dependent manner (Figure 2) [85]. Despite its discovery in other types of cancer, a crosstalk between HOTAIR and miRNAs is worth exploring in lung cancer because miR-331 and miR-130a are tumor suppressors in lung cancer [113],[114].

### Clinical potentials of HOTAIR in lung cancer

HOTAIR can be explored as a biomarker in lung cancer because its elevated expression in lung tumor tissues is correlated with metastasis, drug resistance, and poor survival in patients with lung cancer (Table 1). For instance, in a cohort of 42 patients with NSCLC, 5-year post-operative survival in 21 patients with high expression of HOTAIR is at only 20% vs a 45% survival rate in 21 patients with low expression of HOTAIR [23]. In another cohort of 35 patients with SCLC average disease-free survival is at 30.8 months in 12 patients with high expression of HOTAIR vs average survival of 46.3 months in 23 patients with low expression of HOTAIR [21].

HOTAIR’s feasibility as a biomarker is enhanced by the findings that lncRNAs are stable and measurable in body fluids and thereby suitable for measurement via non-invasive procedures [7]. HOTAIR along with several other lncRNAs can be quantitatively measured in plasma samples collected from patients with gastric cancer [115]. HOTAIR’s power as a biomarker is further enhanced when it is measured in combination with other critical regulators of lung cancer. A combined measurement of exosomal miR-21 and HOTAIR yields greater sensitivity and specificity in distinguishing laryngeal squamous cell carcinoma from benign polyps than each individual measurement alone [77]. This approach can be readily applied to lung cancer because miR-21 is a miRNA signature of NSCLC and co-upregulated by Col-1 in lung cancer cells [19],[99],[116]. Another approach to increase the predictive power of HOTAIR is simultaneous measurement of HOTAIR and its protein partners, e.g., EZH2. As exemplified in a breast cancer study simultaneous increase of HOTAIR and PRC2 has a greater correlation with poor survival than the increase of each marker alone [52].

HOTAIR is an appealing therapeutic target because inhibition of HOTAIR exhibits promising anti-tumor efficacy in preclinical models of lung cancer (Table 1). Moreover, pharmacological inhibitors of PRC2 exhibit convincing anti-tumor efficacy in preclinical models of NSCLC and SCLC [117],[118]. It is important to specifically disrupt the interaction between HOTAIR and PRC2 in cancer cells upon successful molecular and biochemical resolution of the interaction between HOTAIR and PRC2. This approach can potentially spare any HOTAIR-independent physiological functions of PRC2. Another appeal of HOTAIR as a therapeutic target arises from its critical role in resistance to chemotherapy drugs in lung cancer cells [22]. A combination of traditional chemotherapy and inhibition of HOTAIR can potentially overcome drug resistance and increase tolerance to traditional chemotherapy.

### Challenges and future directions

HOTAIR has emerged as a promising diagnostic and therapeutic target for lung cancer (Table 1). However, several challenges hinder realization of HOTAIR’s potential in intervention of lung cancer. One challenge is our limited understanding of the interaction between HOTAIR and its protein partners [41]. A high-resolution map of HOTAIR-PRC2 and HOTAIR-LSD1 interactions is essential to develop compounds that can effectively and specifically disrupt their interaction in lung cancer cells. This is highlighted by the fact that PRC2 physically interacts with thousands of lncRNAs, and its function is tightly regulated by these interacting lncRNAs [119],[120]. It is conceivable that PRC2 forms a pool of functional units as defined by their lncRNA partners, and this pool of PRC2-lncRNA units is dynamically fine-tuned to maintain an appropriate gene expression program to meet the cell’s needs in a particular cellular context. How an increased expression of HOTAIR disturbs this fine-tuned pool of PRC2-lncRNA units and promotes cancer is a daunting question to answer. One can speculate that increased HOTAIR binding to PRC2 can interfere with formation of other PRC2-lncRNA units through competitive binding or alteration of PRC2 conformation. This is critical to lung cancer because TUG1, also a PRC2-interacting lncRNA, exerts its tumor suppressive action through PRC2-mediated repression of HOXB7 [38].

Another challenge arises from EZH2-mediated methylation of non-histone proteins. Undoubtedly, inhibition of either HOTAIR or EZH2 hinders progression of lung cancer (Table 1) [117],[118]. However, the experimental designs in these studies are not able to exclude the possibility that the altered gene expression and cell behaviors can be, at least in part, attributed to altered methylation of transcription factors and other non-histone proteins methylated by EZH2. For instance, EZH2 directly methylates transcription factor GATA4 and diminishes GATA4’s transcriptional activity [121],[122]. HOTAIR-regulated EZH2-dependent methylation of non-histone substrates in lung cancer cells can be explored using a proteomic survey of the methylated proteins with or without inhibition of HOTAIR and/or EZH2.

It is also naive to conclude that PRC2, LSD1, and the newly discovered E3 ubiquitin ligases are the sole protein partners of HOTAIR to mediate its functions in lung cancer [41],[90]. lncRNAs reside in every subcellular compartment and act in a wide range of cell processes, such as signaling transduction, RNA splicing, and ligand-receptor engagement [4]. The versatility of HOTAIR function in lung cancer needs to be explored with a thorough screening of HOTAIR-bound protein partners using HOTAIR as bait in lung cancer cells.

### Summary

HOTAIR has emerged as a novel master regulator of lung cancer. HOTAIR possesses tremendous diagnostic and therapeutic potentials in intervention of lung cancer. Materialization of HOTAIR’s clinical potential requires further investigation of the molecular mechanisms underlying the tumor-promoting actions of HOTAIR in lung cancer.

## Authors’ original submitted files for images

Below are the links to the authors’ original submitted files for images. Authors’ original file for figure 1 Authors’ original file for figure 2

## Abbreviations

lncRNA: Long non-coding RNA
HOTAIR: HOX transcript antisense RNA
PRC2: Polycomb repressive complex 2
EZH2: Enhancer of zeste homolog 2
LSD1: Lysine-specific demethylase 1
PTEN: Phosphatase and tensin homolog
