# Supplementary figures and images for: Functions of lncRNA HOTAIR in lung cancer
Source: J Hematol Oncol. 2014 Dec 10;7:90. doi: 10.1186/s13045-014-0090-4 (PMC4266198; doi:10.1186/s13045-014-0090-4)

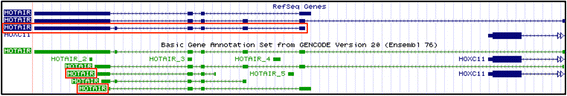

Supplement: Supplementary file 1 — Authors’ original file for figure 1 [file 13045_2014_90_MOESM1_ESM.gif]

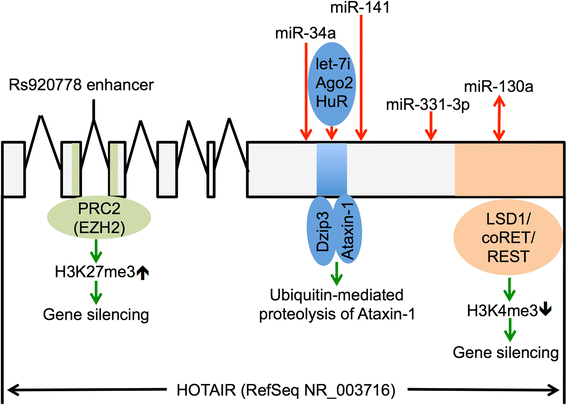

Supplement: Supplementary file 2 — Authors’ original file for figure 2 [file 13045_2014_90_MOESM2_ESM.gif]
